# Acute radiation dermatitis in breast cancer: topical therapy with vitamin E acetate in lipophilic gel base

**DOI:** 10.3332/ecancer.2010.190

**Published:** 2010-12-23

**Authors:** S Martella, M Rietjens, V Lohsiriwat, R Lazzari, A Vavassori, BA Jereczek, V Lazzati, MC Leonardi, JY Petit

**Affiliations:** 1 Departments of Plastic Surgery; 3 Departments of Radiotherapy, European Institute of Oncology, via Ripamonti 435, 20141, Milan, Italy; 2 Siriraj Hospital Mahidol University, Bangkok, Thailand; 4 University of Milan, Milan, Italy

**Keywords:** Radiation dermatitis, breast cancer, vitamin E

## Abstract

**Background:**

Radiotherapy can cause adverse skin reactions over the course of their treatment. Currently, management is based on several tropical products although there is no gold-standard approach to prevention and management of radiation toxicity.

**Method:**

We report our experience of vitamin E acetate in the treatment of radiation dermatitis in breast cancer patients who experienced grade 4 side effects (according to Radiation Therapy Oncology Group criteria).

**Results:**

Clinical management consisted of oral antibiotics and local application of vitamin E acetate and local escarectomy. All of the patients achieved complete re-epithelialization within 40 days.

**Conclusion:**

Skin ulceration and necrosis post-radiation may interrupt oncological treatment in breast cancer patients. In acute radiodermatitis with skin necrosis, we propose the use of oral antibiotics together with escarectomy and the application of vitamin E acetate to facilitate the healing process in order to minimize the interruption to the oncological treatment.

## Background

The standard treatment for early breast cancer includes conserving breast surgery followed by radiation therapy and systemic treatment. One of the common side effects of irradiation is a skin reaction, including acute erythema, desquamation and, rarely, delayed radiodystrophy or radionecrosis. Mild to moderate acute dermatitis occurring during the treatment course is common. While more severe acute injury is rare and normally only occurs in cases with specific skin sensibility or to an area receiving a higher dose of radiation, it may lead to local infection associated with skin ulceration similar to a severe skin burn [1–5]. Severe acute dermatitis may require interruption of the radiation therapy and oncological treatment course. Local treatment of acute radiation dermatitis usually includes corticoid creams [6–8]. We present a case of acute dermatitis occurring during a course of radiotherapy for conservative treatment of breast cancer. The aim of this report is to focus on the local treatment (vitamin E acetate in lipophilic gel (named as Vea Oil ®) plus escarectomy and oral antibiotic administration.

## Method

### Case report:

**Figure 1:**
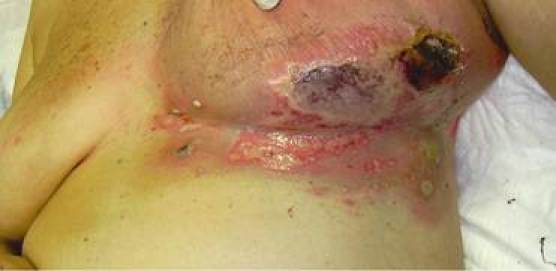
Erythema associated with flyctena, cutaneous necrosis, pain, fever and staphylococcus aureus infection.

A 67-year-old women was submitted to conservative surgery for a ductal infiltrating carcinoma of the left breast, stage pT1c, pN0, M0, G3, ER: 0%; PgR: 0%; Ki-67: 70%; c-erb B2: absent. Post-operative therapy included chemotherapy (CMF - 6 cycles) and external radiotherapy (50 Gy with an additional boost of 10 Gy in tangential fields with photon energy of 6 MV given in 2 Gy daily fractions, 5 fractions/week). The radiotherapy started after the third cycle of chemotherapy, despite the neutropenia. After 40 Gy of radiotherapy, the patient presented a skin lesion of the 4° grade (criteria of the Radiation Therapy Oncology Group, RTOG) erythema, cutaneous necrosis, pain, fever and a Staphylococcus aureus infection (Figure 1). The radiation treatment plan was reviewed and an overdose area was revealed. In this case, local treatment with vitamin E acetate as a lipophic gel, escarectomy and oral antibiotics were administered.

## Result

**Figure 2:**
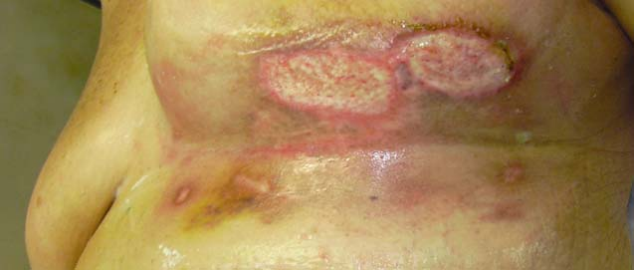
After escarectomy in local anaestesia.

**Figure 3:**
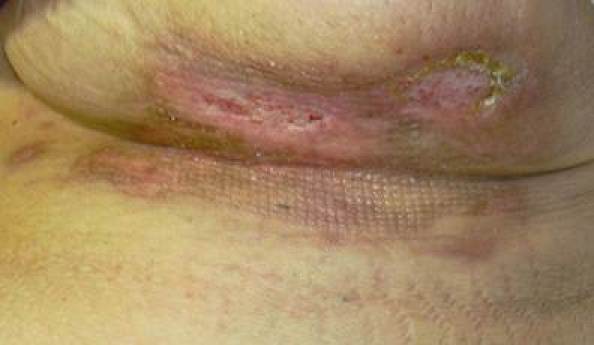
After 25 days of treatment.

**Figure 4:**
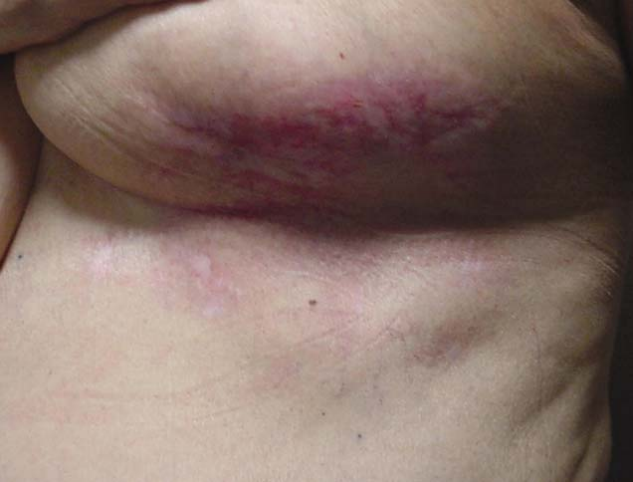
After 40 days of treatment.

This patient was treated with oral antibiotics and local application of vitamin E acetate from the first day after the presentation of the skin lesion. After six days, an escarectomy was performed under local anaesthesia (Figure 2). After 25 days, the infection had resolved and total re-epithelialization was observed (Figure 3). After 40 days, the result was excellent. Further patients were treated with the same procedure, and in all cases, their skin complications healed within 40 days and the same satisfactory results were achieved (Figure 4).

## Discussion

Extensive radiation dermatitis usually requires several months to heal and often does not heal spontaneously. Some of the healed aesthetic results are unsatisfactory and may require plastic surgery procedures. In our case, the association of oral antibiotics and local treatment with Vea Oil®—vitamin E acetate allowed complete healing of the wounds within 40 days. The idea of using the oil alpha-tocopherol acetate for topical skin treatment was based on the following evidence: (a) the redox chemistry of the vitamin, compatible with an anti-oxidant effect *in vivo* [9,10]; (b) the hydrolysis of the ester in the skin [11] and thus the pro-drug nature of the acetate; (c) the prospect of a physiological extracellular function of vitamin E, which is, indeed, secreted and reabsorbed by the skin [12]. The beneficial effect, in relation to the preservation of the barrier function of the skin, is clearly evident.

## Conclusion

Skin ulceration and necrosis post-radiation may interrupt oncological treatment of the breast cancer patient. In acute radiodermatitis with skin necrosis, we propose the use of oral antibiotics together with escarectomy and the application of vitamin E acetate to facilitate the healing process in order to minimize the interruption to the oncological treatment.
